# Detection of Helicobacter Pylori in Nasal Polyps: An Epidemiological Study

**DOI:** 10.1007/s12070-023-03585-9

**Published:** 2023-03-03

**Authors:** Giolanta Zika, Fotios S. Fousekis, Georgios Exarchakos, Gerasimos Baltayiannis

**Affiliations:** 1 Department of Gastroenterology, School of Health Sciences, Faculty of Medicine, University Hospital of Ioannina, University of Ioannina, Ioannina, Greece; 2 Department of Otorhinolaryngology, Head and Neck Surgery, School of Health Sciences, Faculty of Medicine, University Hospital of Ioannina, University of Ioannina, Ioannina, Greece; 3 Department of Gastroenterology and Hepatology, School of Health Sciences, Faculty of Medicine, University Hospital of Ioannina, University of Ioannina, PO Box 1186, 45110 Ioannina, Greece

**Keywords:** Nasal polyps, Helicobacter pylori, Sinusitis, Rhinosinusitis

## Abstract

Many studies have described the detection of Helicobacter pylori (HP) in the nasal polyps; however, although gastroesophageal reflux has been associated with chronic rhinosinusitis and nasal polyps development, the role of HP remains unclear. Our aim was to describe the prevalence of HP detection in nasal polyps and its association with gastric HP infection and gastroesophageal reflux dsease (GERD). The prospective study involved 36 patients with nasal polyps, who underwent to endoscopic surgery removal of nasal polyps. Before surgical procedure all patients were tested for gastric HP infection by ^13^ C-urea breath test, while tissue samples from nasal polyps were tested for HP detection, using rapid urease test (CLO test) and histological examination with Giemsa stain. All patients were asked about GERD-related symptoms. HP in nasal polyps was detected in 9 out of 36 patients (25%) using histological examination with Giemsa stain, while the detection rate of HP was 30.5% (11/36) using CLO test. Furthermore, 28 out of 36 patients (77.7%) had gastric HP infection. All patients with HP colonization in nasal polyps had gastric HP infection and all patients with HP in nasal polyps reported symptoms related to GERD. HP was detected in approximately one out of three patients in nasal polyps, while all patients with HP detection in nasal polyps had concurrently gastric HP infection and reported GERD-related symptoms, suggesting a gastro-nasal route of HP.

## Introduction

Helicobacter pylori (HP) is a gram-negative, spiral and microaerophilic bacterium that was firstly identified in 1982 by Robbin Warren and Barry Marschal. HP is mostly detected in stomach and HP infection has been associated with both gastric diseases, such as, gastritis, peptic and duodenal ulcer development, gastric cancer and MALT lymphoma, and extra-gastric diseases, such as idiopathic immune thrombocytopenic porpura and rosacea [1]. Gastric HP infection may be confirmed by various methods, including, urease breath test, histological examination of gastric tissue with histochemical methods and/or immunohistochemistry, rapid urease test (CLO), stool antigen test and antibodies detection [2]. The estimated prevalence of gastric HP infection ranges between 30% and 45% in developed countries and is higher than 50% in developing countries [3].

Except the stomach, HP colonization has been documented in multiple sites within the body, such as middle ear, oral cavity, gallbladder and large intestine [4]. Moreover, many studies have demonstrated the detection of HP in nasal cavity and nasal polyps, suggesting a possible role of HP on development of chronic rhinosinusitis and nasal polyposis [5]. Nasal polyps are noncancerous, benign hyperplastic growth of nasal mucosa and may cause a wide spectrum manifestations, including nasal obstruction, anosmia and rhinorrhoea [6], affecting from 1 to 4% of the general population. The etiology of nasal polyps has not been fully clarified and several conditions, including bronchial asthma, chronic rhinosinusitis, eosinophilic granulomatosis with polyangitis and cystic fibrosis, have been associated with nasal polyposis. The management of nasal polyposis may be both medical and surgical [7].

The purpose of this study is to demonstrate the prevalence of HP in nasal polyps by different methods, using rapid urease test (CLO test) and histological examination with Giemsa stain. Furthermore, a breath test was performed in all subjects with nasal polyps in order to examine the association between nasal and gastric HP colonization. In addition, a great emphasis has been placed on presentation and analysis of epidemiology, transmission and the possible role of HP on nasal polyps.

## Study Design and Material

Our study was prospective and was conducted at University Hospital of Ioannina from December 2017 to April 2020. We included 36 adult patients with nasal polyps, who underwent to endoscopic surgery removal of nasal polyps. Indications of procedure were recurrent sinusitis, nasal obstruction and continued symptoms despite medical management. Sample tissue from resection nasal polyps was tested for HP detection, using rapid urease test - campylobacter like organisms (CLO test) and histological examination with Giemsa stain. In addition, before surgical procedure all patients were tested for gastric HP infection by ^13^ C-urea breath test. Every patient was asked about gastroesophageal reflux disease (GERD)-related symptoms, such as heartburn and regurgitation.

Moreover, informed consent was taken in all patients before endoscopic procedure, while exclusion criteria of study were taking antibiotics in the last month before surgery, proton pump inhibitors in the last ten days and/or receiving therapy for HP eradication in the past.

## Diagnostic Methods

Regarding to diagnostic methods, the histological Giemsa-staining examination involved the following stages: Initially, the deparafinnized histological sections were embedded in Giemsa solution for 30 min. Then, the sections were embedded in acetic acid and after in ethanol 96°. Thereafter, the histological specimens were embedded in propanol for 2 min and then in xylene for 2 min. All slides were examined at ×400 by two pathologists and a positive result was considered the presence of microorganisms with morphological characteristics compatible with HP in the nasal mucosa.

In addition, the tissue specimens from nasal polyps were placed in CLO test kits. After 30 min, 3 and 24 h, we examined the color of Kit and orange or red color was recorded as a positive result.

### Statistical Analysis

Data were analyzed using SPSS software version 22. All data were categorized as either categorical or continuous variable and continuous variables were summarized as means and standard deviation.

## Results

**Fig. 1:**
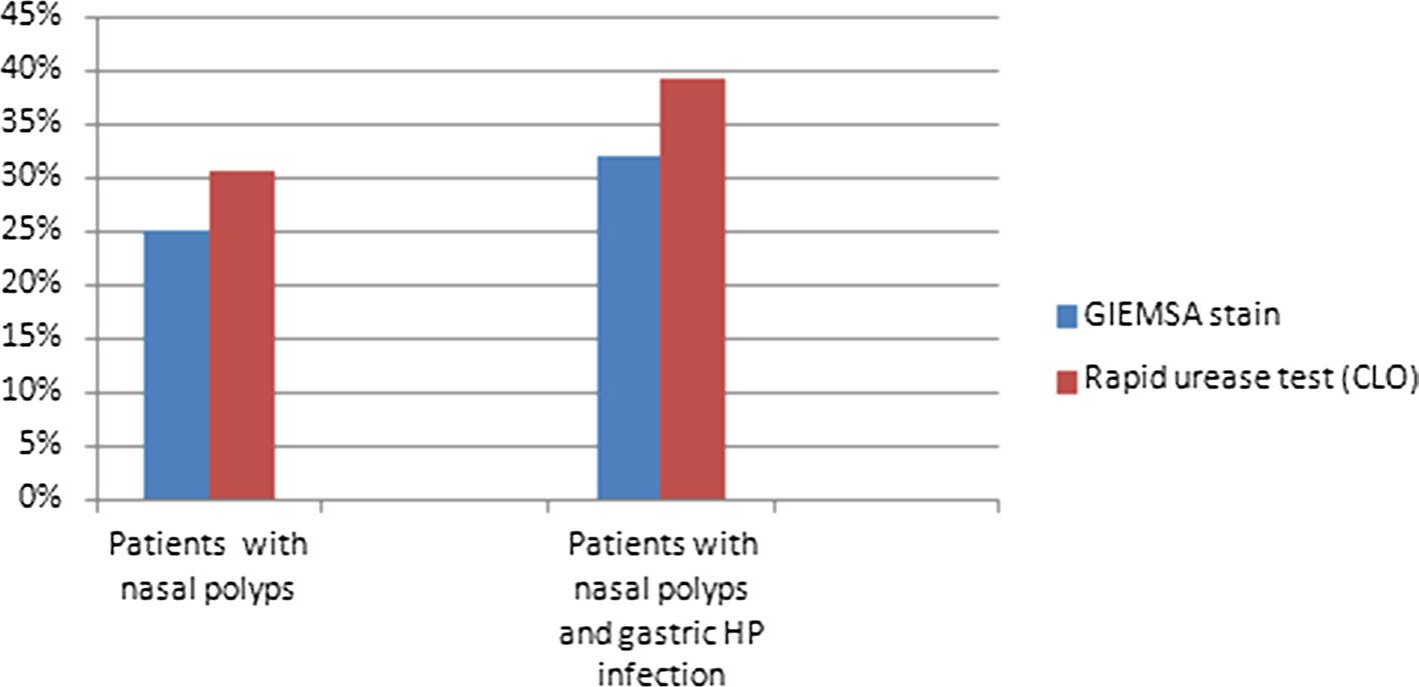
Helicobacter pylori organisms in nasal polyp (Giemsa stain ×400)

**Fig. 2:**
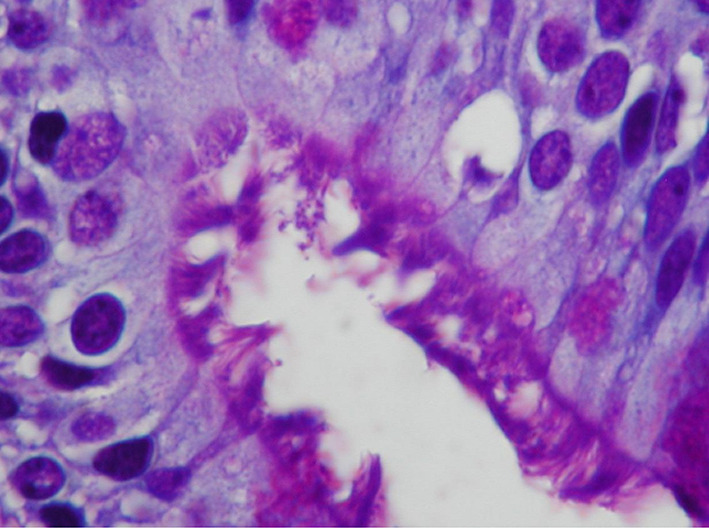
Prevalence of HP colonization in nasal polyps overall and in nasal polyps of patients with gastric HP infection

The average age of patients was 61 years (SD 5) at time of polypectomy. 69.4% (25/36) of patients were males and 30.6% (11/36) were females. In nasal polyps, HP was detected in 9 patients (25%) using histological examination with Giemsa stain (Fig. 1), while the frequency rate of HP was higher (11/36, 30.5%) using CLO test. With regard to gastric HP colonization, 28 out of 36 patients (77.7%) had positive ^13^ C-urease breath test. All patients with HP colonization in nasal polyps had gastric HP infection, while all patients with HP detection in nasal polyps reported symptoms related to GERD. The frequency rate of HP in nasal polyps among patients with gastric HP infection was 32.1% (9/28) using histological examination and 39.2% (11/28) using rapid urease test (Fig. 2).

## Discussion

In our study, we detected HP colonization in nasal polyps and the prevalence ranged from 25 to 30% depending on the diagnostic method, while higher detection rate was demonstrated using rapid urease test. The frequency rate of HP detection in nasal polyps was similar to many studies (Table 1) [8–17].

**Table 1:** Studies about prevalence of Helicobacter pylori in nasal polyps

Study	Origin of study	Study design	Number of patients	Diagnostic method	Results
Vceva et al. [8]	Croatia	Prospective	35	PCR ELISA	28.5% (10/35) 85.7% (30/35)
Nemati et al. [9]	Iran	Prospective	25 patients without GERD symptoms	PCR Urease test Culture	0% 0% 0%
Burduk et al. [10]	Poland	Prospective	20	PCR (ureA gene) PCR (cagA gene)	100% (30/30) 0%
Ozyurt et al. [11]	Turkey	Prospective	32	Real-time PCR	59.4% (19/32)
Ozcan et al. [12]	Turkey	Prospective	25	CLO test Immunohistochemistry ELISA	4% (1/25) 0% 24% (6/25)
Cvorovic et al. [13]	Serbia	Prospective	23	Urease test Giemsa staining	26% (6/23) 26% (6/23)
Szczygielski et al. [14]	Poland	Prospective	61	Urease test	0%
Koc et al. [17]	Turkey	Prospective	30	ELISA Immunohistochemistry	86.7% (26/30) 20% (6/30)
Bansal et al. [15]	India	Prospective	35	Immunohistochemistry	40% (14/35)
Siupsinskiene et al. [16]	Lithuania	Prospective	45	Immunohistochemistry & urease test	28.9% (13/45)
Jelavic et al. [18]	Bosinia and Herzegovina	Prospective	40	Immunohistochemistry	70% (28/70)

We performed an in-depth review of the literature in PubMed to identify articles about epidemiology of HP in nasal polyps, using the following search string: (“Helicobacter pylori”) AND (“nasal polyps” OR “nasal polyposis” OR “sinusitis”). The frequency rate of HP in nasal polyps is varied depending on method of detection. The prevalence of HP colonization ranges from 0 to 100%. The most studies demonstrated a frequency rate between 25 and 60%, while only one study documented no HP colonization in nasal polyps in patients without GERD symptoms, suggesting a gastro-nasal route of HP [9]. No association between HP colonization in nasal polyps and other factors, such as age, gender, presence of allergy, nasal side or co-existence tonsillar disease has been determined [16]. In addition, a study described that HP sinonasal colonization has no prognostic value for efficacy of functional endoscopic sinus surgery on chronic rhinosinusitis symptoms, but presence of HP was associated with greater improvement of the postoperative endoscopic scores [18]. Furthermore, prevalence seems to be higher using ELISA method for HP detection. In our study, rapid urease test and histochemical examination with Giemsa stain were performed and the most cases of HP colonization demonstrated by urease rapid test. It is worth mentioning, that there are many urease-producing bacteria both in the oral cavity, such as Streptococcus salivarius and Actinomyces naeslundii [19] and urease pathogens in lungs, such as mycobacteria [20], which may colonize the polypoid tissue, producing false positive urease results in urease test and causing higher HP detection rates using CLO test compared to histologic examination with Giemsa stain. However, Giemsa staining is not affected by urease containing organisms.

There are two transmission routes of HP: oral-oral transmission and fecal-oral transmission. The mechanism of transmission and colonization of HP in sinonasal cavity has not been clarified; however, three possible modes have been suggested. First, nasal cavity may be a reservoir of HP. Second, many studies have been described HP colonization in the oral cavity that may be a reservoir of HP [21], which has been detected in dental plaque, tonsillar and adenoid tissue [22, 23]. Therefore, HP may be transmitted to nasal cavity by oronasal reflux. Third, HP colonizes the stomach, which is a reservoir of HP and the bacteria are transmitted to the sinonasal cavity by gastroesophageal reflux [15]. A prospective study documented high rate of HP colonization (75%) in nasal polyp of patients with GERD and gastric HP infection, suggesting a strong association between HP infected nasal polyps and GERD [13]. In our study, all patients with HP in nasal polyps reported GERD symptoms and suffered from gastric HP infection, suggesting transmission of HP from stomach to nasal cavity.

However, it is worth mentioning that HP colonization of nasal polyps in patients with GERD may probably support a reservoir thesis similar to other extragastric sites, because many studies have suggested that gastroesophageal and laryngopharyngeal reflux are linked to nasal disorders and may contribute to chronic rhinosinusitis development via gastric acid exposure and triggering inflammation pathways [24]. A population-based study from Taiwan documented that patients with GERD-related symptoms carry a higher risk of chronic rhinosinusitis development compared with patients without GERD (HR: 2.36; 95% CI  2.08–2.68; *P* < 0.001) [25]. Moreover, a control study investigated the role of gastroesophageal and laryngopharyngeal reflux on sinusitis and development of nasal polyps, detecting the expression of pepsin in nasal cavity of patients with chronic sinusitis with or without nasal polyps and in patients without sinusitis. They found association between reflux and chronic sinusitis, while laryngopharyngeal reflux was associated with severity of nasal polyps [26].

On the other hand, the role of HP on chronic sinusitis and nasal polyposis development is still unclear. In the stomach, many pathogenic mechanisms have been proposed about HP-induced inflammation. HP may trigger the production of inflammatory cytokines, chymokines and growth factors via different virulence factors, such as, CagA and VacA, leading to recruitment of immune cells to the lamina propria in the stomach [27]. Thus, HP colonization of nasal may be associated with chronic inflammation, contributing to nasal polyposis development; however, the data is limited and several studies have suggested a disputed association between HP and development of nasal polyps. A prospective study demonstrated that HP colonization in nasal cavity was more prevalent in patients with chronic sinusitis compare to control subjects. Furthermore, in this study, the severity of chronic rhinosinusitis was evaluated according to Computer Tomography findings using Lund-MacKay scoring system, demonstrating no association between intranasal HP colonization and severity of rhinosinusitis and concluding no role of HP on pathogenesis of sinusitis and nasal polyposis [28]. Thus, it may be assumed that gastric juice carrying HP refluxes into nasal cavity and not HP itself contributes to rhinosinusitis and nasal polyposis [29]. A recent study investigated the role of gastric HP infection on nasal mucociliary clearance. It demonstrated that patients with gastric infection had increased nasal mucociliary clearance time compared to HP-negative patients, while the nasal mucociliary clearance time was normalized after eradication of gastric HP eradication, proposing that HP infection may have an essential role on the chronic sinusitis development. However, a major limitation of this study was the lack of examination for nasal HP colonization [30]. Consequently, further investigation is needed.

In our study, there were a few of limitations. The sample of patients was small and a control group without nasal polyps did not exist. Also, the gold standard of diagnosis of gastric HP infection is the gastric biopsies and we performed breath test for HP detection in the stomach. Although PCR present more sensitivity and specificity for HP detection, we performed histochemical examination and CLO test.

## Conclusion

In our study, HP colonization in nasal polyps was detected in one third of patients, while all patients with HP positive had GERD symptoms and gastric HP infection, suggesting a gastric-nasal route of HP. However, the association between gastroesophageal reflux and chronic sinusitis with nasal polyps has documented in many studies and there is no clear evidence about the contribution of HP nasal colonization to nasal inflammatory status and development of polyps. Therefore, the role of HP on sinusitis and nasal polyps’ development are controversy and further investigation is warranted.

## Data Availability

Data was generated at the University hospital Ioannina, Greece. The data that support the fndings of this study are available on request from the corresponding author.
